# Supplementary material for: MetaRibo-Seq measures translation in microbiomes
Source: Nat Commun. 2020 Jun 29;11:3268. doi: 10.1038/s41467-020-17081-z (PMC7324362; doi:10.1038/s41467-020-17081-z)
Supplement: Supplementary file 10 — Supplementary Data 7 [file 41467_2020_17081_MOESM10_ESM.zip › File2/Confidence_High_Taxonomy/186566_out.krona.html]

Javascript must be enabled to view this page.

members
magnitude
magnitudeUnassigned
count
unassigned
taxon
rank

186566\_out

3

superkingdom
2759
3

kingdom
4751
3

subkingdom
451864
3

phylum
3
4890

3
147538
subphylum

3
147550
class


SRS1041145\_contig\_number\_36516
subclass
1
222543
3

1028384
2
order

2
1033978
family

2
1036719
genus


SRS023914\_contig\_number\_contig-100\_531.156992SRS053356\_contig\_number\_contig-100\_1514.246385
1051613
2
species
